# Effects of COVID-19 on maternal institutional delivery: Fear of a rise in maternal mortality

**DOI:** 10.7189/jogh.11.03041

**Published:** 2021-03-01

**Authors:** Md Ashfikur Rahman, Henry Ratul Halder, Sheikh Mohammed Shariful Islam

**Affiliations:** 1 Development Studies Discipline, Khulna University, Khulna 9208, Bangladesh; 2 Department of Community Health Sciences, University of Manitoba, Winnipeg, Manitoba, Canada; 3 Institute for Physical Activity and Nutrition, School of Exercise and Nutrition Sciences, Faculty of Health, Deakin University, Burwood, Victoria, Australia

Since the outbreak of the novel coronavirus or SARS-CoV-2 (COVID-19), the word has witnessed multidimensional problems and challenges. The global social, economic and public health sectors are more vulnerable and going through an unprecedented crisis. During this overwhelm pandemic, maternal mortality could be exacerbated due to the redirection of health care services towards COVID-19. The continuous lockdown, fear of getting infected with COVID-19 and massive disruption in the provision of maternal health services (such as antenatal and postnatal care) has resulted in a significant decline in the institutional delivery rate in Bangladesh [1]. These disruptions could push back many low-and-middle-income countries (LMICs) endeavour on ensuring safe institutional deliveries.

Globally, approximately 810 maternal deaths occur every day, most of which are preventable by using institutional and safe delivery [2]. In 2019, about 80 million deliveries occurred at health institutions globally [3], but this number may be reduced in a post-pandemic scenario. Pregnant women who deliver at home have an increased risk of maternal mortality due to factors such as haemorrhages, eclampsia, sepsis, and obstructed labour, etc. [4,5]. Evidence shows that 35% of all causes of antepartum, intrapartum and postpartum haemorrhage is due to unsafe home delivery practices [4-6]. Between 2010 and 2017, the maternal mortality ratio (MMR) in Bangladesh decreased substantially to 173 per 100 000 live births. Using institutional delivery with skilled health care professionals could reduce 16 to 33% of maternal deaths, globally [7,8]. Still, during this pandemic, the rate of institutional deliveries has been declining overwhelmingly. A study published in The Lancet [3] pointed out that institutional childbirth reduced by more than half in Nepal. A similar picture was presented for India [9] due to the lack of transport, fear among people and doctors, resulting in thousands in need of health care services being denied by hospitals.

Before COVID-19 outbreak, half of the pregnant women in Bangladesh preferred to give birth at home, but the rate raised to 73% following the announcement of the lockdown on 26 March 2020. The pre-post lockdown differences are equally stark in the case of caesarian section [10]. District-wide data from Bangladesh confirms the worrying dip for institutional delivery [10]. Almost 20% of declines were found in most districts for both normal delivery (43 districts) and caesarian section (40 districts). In comparison, about one-third of the districts observed higher drops up to 40% for normal delivery in 19 districts and caesarian section in 18 districts. Major disruptions in institutional delivery occurred in the capital city Dhaka for both normal delivery at 42% and c-section at 41% [10].

**Figure:**
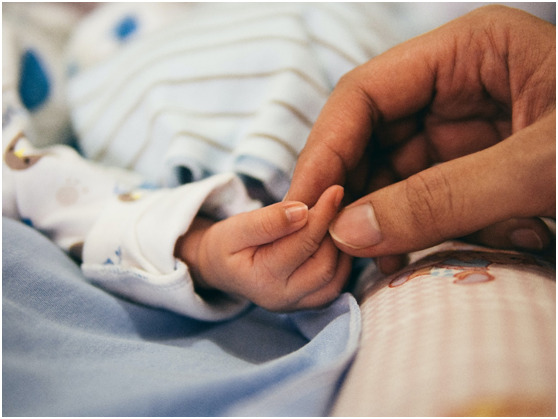
Photo: Mother is holding her baby’s hand. The photo was taken from a licence-free online source which (https://unsplash.com/photos/5zp0jym2w9M).

The Millennium Development Goals 1990-2015 emphasised the importance of reducing maternal and child mortality by 75%. It resulted in the considerable reduction of maternal mortality rate to 38% worldwide [11]. The Sustainable Development Goals 3 (SDGs) targets to reduce MMR to less than 70/100 000 per live births [12]. If the increasing trend of home delivery continues, it would be impossible for many LMICs, including Bangladesh, to meet the SDGs target. Other emerging concerns due to COVID-19 lockdown include increased food and wealth insecurity in households [13] that might reduce the capacity to avail the essential services from hospitals for childbirth or antenatal care. At the same time, institutional deliveries and caesarian section provided by the private sector in many LMICs have either shut or refused to provide services. These disruptions highlight the shortcomings of the fragile and unplanned health system and raise questions on the appropriateness of the health policies and programs.

Urgent measures are needed to continue to provide high-quality maternal health services during and after COVID-19 pandemic. These include but are not limited to develop special interventions-for the pregnant women for any kinds of emergency; establish trust between communities and individuals, particularly frontline health care providers; providing safety equipment, such as personal protective equipment and financial incentives for any undesirable uncertainties. Further, there is a need to monitor and routinely check-the existing facility service in different hospitals levels from local to community and private clinics. Regulations and strict enforcements are essential so that health care institutions cannot deny providing essential maternal services. Training for the health workers – including doctors, nurses and midwives through online can be initiated to provide antenatal care to patients over mobile phones or any online platforms. Finally, in-depth research on pregnant mothers and service providers can be incepted to look into both supply and demand factors and to sort out the barriers to maternal health services amid COVID-19. Special attention should be provided to address panic and anxieties about visiting health care facilities along with the behavioural change of the health workers.
